# Evaluation of Mercury Exposure Reduction through a Fish Consumption Advisory Program for Anishinaabe Tribal Members in Northern Wisconsin, Michigan, and Minnesota

**DOI:** 10.1155/2010/802584

**Published:** 2010-07-25

**Authors:** J. A. Foran, A. D. DeWeese, M. J. Hudson, N. E. Kmiecik

**Affiliations:** ^1^EHSI, LLC, Whitefish Bay, 5005 N. Palisades Rd., WI 53217, USA; ^2^Wisconsin Department of Natural Resources, Madison, WI 53707, USA; ^3^Bad River Watershed Association, Ashland, WI 54806, USA; ^4^Great Lakes Indian Fish and Wildlife Commission, Odanah, WI 54861, USA

## Abstract

The Great Lakes Indian Fish and Wildlife Commission has an extensive program to inform Anishinaabe tribal members from northern Wisconsin, Michigan, and Minnesota who harvest and consume walleye about the health risks of consuming these fish, and to encourage harvest and consumption practices that reduce exposure to MeHg. We report here the results of a probabilistic analysis of exposure to methyl mercury (MeHg) among tribal members who consume walleye. The model predicts that the potential for greatest exposures to MeHg occur among women of child-bearing age and children who consume large walleye from lakes that contain heavily contaminated (MeHg concentration >0.5 mg/kg) fish. The analysis allows GLIFWC to evaluate, focus, and fine-tune its initiatives to protect the health of tribal members in ways that result in exposure and risk reduction for tribal harvesters, women of child-bearing age, and children, while maintaining important tribal lifeways, which include the harvest and consumption of walleye.

## 1. Introduction

The effects of exposure to low levels of methyl mercury (MeHg) are well documented and include developmental deficits, particularly in children exposed prenatally [1, 2]. A significant source of MeHg in the US diet is the consumption of contaminated fish, and in 2004 the USFDA and USEPA issued a joint announcement advising women of child-bearing age, pregnant women, and young children to avoid consumption of shark, swordfish, tilefish, and mackerel, and to limit the consumption of albacore tuna [3]. Many sport or subsistence-caught freshwater fish species also contain elevated levels of MeHg and, as a result, state and tribal organizations in the Great Lakes basin and elsewhere issue advice to reduce or avoid consumption of these fish [4–9]. 

Native Americans often consume greater quantities of freshwater fish than the general public [10] and, therefore, may be exposed to higher levels of MeHg. Anishinaabe (Ojibwe or Chippewa) in the Great Lakes region (hereafter—tribal members), including those who belong to tribes that are members of the Great Lakes Indian Fish and Wildlife Commission (GLIFWC), harvest and consume freshwater fish as part of their traditional lifeways, an approach to living that incorporates culture, spirituality, language, and traditions including consumption of indigenous foods. Historically, fish comprised 17–38% of the traditional diet of Anishinaabe in Northern Wisconsin [11]. Walleye (*Sander vitreus*), a top predator that has elevated tissue concentrations of MeHg, is the species most frequently harvested and consumed by tribal members. Most walleye harvesting and associated consumption occur in the spring of the year following ice-out conditions on inland lakes. In spring 2006, approximately 75,800 adult walleye were harvested from 191 inland lakes in the 1837 and 1842 ceded territories of Michigan, Minnesota, and Wisconsin [12–14]. Because of harvest and consumption characteristics, tribal members in the Northern Great Lakes region may be exposed to elevated concentrations of MeHg and, as a result, GLIFWC issues advice that encourages behavior that reduces exposure to MeHg associated with harvest and consumption of walleye [9].

GLIFWC develops and disseminates lake-specific, risk-based, culturally sensitive walleye consumption advice via color-coded maps [9, 15]. Color codes correspond with walleye consumption advice (Table 1), with lakes coded blue associated with the least restrictive advice (eat up to 8 meals/month) and red lakes associated with the most restrictive advice (do-not-eat). Advice categories are chosen based on the goals of protecting the health of tribal members (reducing mercury exposure) and preserving tribal lifeways (walleye harvest and consumption). Each advice category constrains consumption of contaminated walleye to levels that limit mercury exposure to the US EPA reference dose (RfD) for methyl mercury. Eight versions of the advisory maps are prepared, one for each of the six GLIFWC-member tribes in Wisconsin, a seventh for select lakes in Minnesota, and the eighth for select lakes in Michigan. 

From 1997 to 2002, GLIFWC conducted a survey to determine fish consumption rates and patterns of tribal members in northern Minnesota, Michigan, and Wisconsin [9]. Consumption data from the survey were combined with data on tribal harvest levels and concentrations of MeHg in harvested fish tissue [15] to model the exposure to MeHg via consumption of contaminated walleye among tribal members. We report here the results of the analysis of MeHg exposure among a group of fish consumers from GLIFWC-member tribes. The results are used to assess GLIFWC's efforts to reduce mercury exposure and health risks associated with consumption of contaminated walleye in ways that maintain the important tribal lifeways of walleye harvest and consumption [9].

## 2. Methods

The objective of the exposure analysis was to identify subgroups of the tribal population with potential for the highest exposures to MeHg from their walleye consumption patterns, and to assess the impact of risk mitigation advice on exposure and risk reduction. Particular emphasis in this study was focused on gender- and age-specific exposure profiles. 

Fish consumption profiles for each gender (male/female) and four age groups (children aged 1–5 years, children aged 6–14 years, women of child-bearing age, and males older than 14 years and females beyond child bearing age) were created from the GLIFWC fish consumption survey (described below and in [9]) using the LifeLine Dietary Record Generator (DRG). The probability that an individual (of a given gender and age) consumed walleye on a given day (including 0 or no fish consumption) and the intake when walleye were consumed were estimated from the fish consumption survey.

The DRG constructs a file of age- and gender-specific dietary records reflecting individual walleye consumption, which serves as the basis for the fish intake parameter of the exposure algorithm; one of the two parameters imported into the exposure/risk assessment software (Customized Dietary Assessment Software—CDAS). Multiyear, multi-site concentrations of MeHg in walleye tissue, the second parameter imported into the CDAS, were arranged into a series of residue concentration distributions, each representing a different lake color code (described below) and fish size category. 

The dietary intake profiles and distributions of MeHg concentrations in walleye tissue were brought together in the CDAS to yield a series of exposure assessments, each representing a different scenario of lake color code and fish size category. The software utilizes a probabilistic approach, drawing a walleye intake value for each “simulated” person (defined by age and gender) and a MeHg concentration value from the residue distribution. Ten thousand iterations were run for each simulated person. The resultant exposure distribution provides the median and 95th percentile exposure values (among others) for selected age/gender groups and can be reported for various lake color code/fish size scenarios. The interindividual exposure variation is captured in the distribution of these 10,000 iterations. 

Changes to the dietary profiles by age and gender resulting from mitigation options (fish consumption advice) were captured in the Dietary Record Generator by creating new dietary records for each mitigation option. Mitigation options addressing the potential residues of MeHg in the consumed fish were reflected by selection of residue concentration distributions from lake color code or fish size categories. The exposure assessments were then rerun using these consumption and residue data files modified as a result of mitigation options.

### 2.1. Walleye Consumption

Walleye consumption data were drawn from a survey of tribal fish consumption conducted by GLIFWC from 1997 to 2002 (additional details of the consumption study are provided in [9]). Fifty-one families, nearly all of which included children under the age of 15, from 10 tribes, recorded their fish consumption in food diaries during the study. Nine to twelve families participated each year, and three families participated during two study years. One family member recorded each participating family member's meals of harvested fish eaten at home over the course of a study year. Meal frequency information was collected for eight months during year 1 (April 1, 1997-November 30, 1997); thus, year 1 data were used in calculations of spring but not annual consumption rates. Fish consumption information, including meal frequency, was collected for 12 months during years 2–5, and these data were used in calculations of seasonal and annual consumption rates and meal size. 

Consumption rates reported here and used in the probabilistic analysis reflect at-home consumption of walleye, but not other fish or fish purchased or consumed away from home (e.g., at restaurants or tribal ceremonies); thus, total fish consumption rates among tribal members are higher. Of the 1699 meal records of harvested walleye eaten at home, 114 (6.7%) were meals of walleye mixed with another species. In these cases, we limited the analysis to walleye by dividing the total grams eaten by the number of species reported for the meal. Approximately 3% of recorded meals contained no meal weight information. A participant's average meal weight was used in these instances. 

The frequency of walleye meals consumed by each participant was determined from the consumption survey for spring (April through June—91 days), summer (July through September—92 days), fall (October through December—92 days), and winter (January through March—90 days). Each participant's seasonal meal frequency was divided by the number of days in each season to obtain the daily probability of consuming walleye in each season.

Consumption probabilities/frequencies and portion sizes were calculated and entered directly into the *LifeLine* software for the corresponding age and season without data fitting or other alterations. While average consumption rates reported below provide a summary of consumption behavior, we analyzed, via *LifeLine*, the entire range of data to preserve and more closely represent actual consumption habits of tribal members. By preserving the actual pattern of consumption among tribal members instead of using average rates, it is possible to investigate the effect of different consumption patterns upon the exposure profile of an entire community. Tribal members consuming small or large amounts of walleye as well as those consuming walleye more or less frequently are visualized by this technique.

### 2.2. Methyl Mercury Concentrations in Walleye Tissue

GLIFWC has a database of 4,555 samples of lake- and size-specific fish tissue concentrations of MeHg generated over 19 years (including years when the fish consumption survey was conducted) from 224 lakes in the 1837 and 1842 ceded territories of Wisconsin, Michigan, and Minnesota. GLIFWC has combined these data with walleye tissue analyses conducted by state agencies and has developed fish consumption advice for 293 lakes in the ceded territories, 207 of which are harvested by tribal members, following Madsen et al. [15] and as described below. 

Lake-specific MeHg concentrations in walleye tissue and walleye lengths were log transformed and used to develop regressions (ln MeHg = slope ∗ Length + intercept) for the 293 lakes with consumption advice developed by GLIFWC [15]. Lake-specific regression equations were applied to individual walleye lengths from each of the 207 lakes harvested by tribal members between 2005 and 2007 to obtain predicted MeHg concentrations for all harvested walleye. Eight predicted concentrations from this analysis exceeded the maximum MeHg concentration in the GLIFWC sampling database (3.10 mg/kg wet weight). While walleye with mercury concentrations greater than 3.10 mg/kg likely exist in some lakes, we capped the predicted concentration in the probabilistic analysis at 3.10 mg/kg to avoid overestimating mercury exposure. To account for different harvest levels from each lake, a distribution of predicted MeHg concentrations was developed and weighted based on the proportion of total annual harvest from each lake. The resulting distributions were entered as residue files in the *LifeLine* software.

GLIFWC analyzes, and tribal members typically consume, skin-off walleye fillets. However, tissue concentration data provided by state agencies and included in the GLIFWC database are drawn from skin-on walleye samples, which are approximately 16% lower than skin-off concentrations [16]; therefore, we converted all skin-on concentrations of MeHg to skin-off concentrations by multiplying skin-on concentrations by a factor of 1.16.

### 2.3. Exposure Analysis

Probabilistic estimates of MeHg exposure were developed for three scenarios: (a) walleye harvested and consumed from all lakes regardless of lake color codes included on GLIFWC advisory maps (described below and in [9]), (b) walleye harvested and consumed from color-coded red lakes (do-not-eat consumption advice), and (c) lakes with color codes other than red-restricted consumption advice. Methyl mercury exposure was evaluated in each scenario for walleye smaller than 41 cm (about 16 inches), walleye larger than 41 cm, walleye smaller than 51 cm (about 20 inches), and walleye larger than 51 cm. Output was analyzed using SAS [17] system for Windows version 6.12 to provide exposure estimates for age-sex groupings beyond those available in the *Lifeline *software. Probabilistic estimates of MeHg exposure were compared with the US EPA [18] reference dose (0.1 ug/kg/day) to provide a qualitative (or semiquantitative) expression of risk.

## 3. Results and Discussion

### 3.1. Methyl Mercury Concentrations in Walleye Tissue

Concentrations of MeHg in tissues of walleye are as high as 3.10 mg/kg wet weight with the greatest concentrations generally occurring in larger fish (Table 2). Walleye consumption advice is developed by GLIFWC for individual lakes based on size- and lake-specific concentrations of MeHg in walleye tissue. The goal of GLIFWC's advisory program is to encourage harvest and consumption of walleye while minimizing exposure to MeHg. Consumption advice is based on MeHg concentrations in tissues of a 51-cm (20 inch) walleye [15], although GLIFWC encourages consumption of smaller fish; therefore, we conducted the probabilistic exposure analysis for walleye larger and smaller than 51 cm as well as fish larger and smaller than 41 cm (16 inches), the mean length of all harvested walleye (Table 3).

### 3.2. Walleye Consumption

Participants in the GLIFWC fish consumption survey consumed harvested walleye at-home at a mean rate of 5.6 g/day (95th percentile 18.0 g/day). At-home walleye consumption rates were greater for males aged 15 years and older (mean 9.1 g/day, 95th percentile 36.3 g/day) than for the other age-sex groups. There was an annual pattern of at-home harvested walleye consumption, with peak rates for participants occurring in the spring concurrent with and following the annual harvest. The mean at-home harvested walleye consumption rate during this period was 10.1 g/day (95th percentile—29.7 g/day). Males aged 15 years and older had the highest at-home consumption rate of harvested walleye during spring (mean—15.0 g/day, 95th percentile—49.2 g/day). Walleye consumption rates for all groups were lower in periods other than spring [9].

### 3.3. MeHg Exposure

GLIFWC evaluated the efficacy of its advisory program [9] and found that it increased awareness of advisory maps among Wisconsin, Michigan, and Minnesota harvesters as well as among women of child-bearing age in Wisconsin (the only state where that group was assessed). The program also resulted in an increase in the percentage of tribal harvesters who preferred smaller walleye (with lower mercury concentrations), although a similar behavioral change did not occur among women of child-bearing age in Wisconsin. Concurrent with these behavioral changes was an increase in tribal walleye harvest, indicating that the advisory program did not adversely affect this important tribal lifeway [9]. However, we did not determine directly whether changes in consumption behavior and MeHg exposure were influenced by the advisory process since the source-lake color codes, walleye length, and MeHg concentrations were not determined for walleye consumed by individuals participating in the consumption survey. Therefore, we modeled MeHg exposure of tribal members who participated in the GLIFWC fish consumption survey by assuming that participants consumed walleye from size distributions similar to the harvest record for each lake depicted on GLIFWC advisory maps. This is appropriate, as walleye are harvested by tribal members, with few exceptions, from a defined (declared) set of lakes, and detailed harvest records are maintained by GLIFWC for these lakes. 

**Figure 1:**
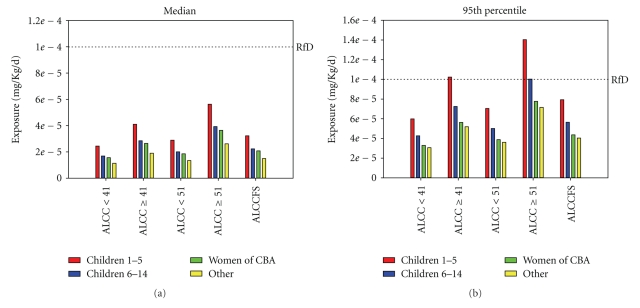
Median (a) and 95th percentile (b) exposure for all lakes/color codes, size. ALCC: all lakes, color codes; ALCCFS: all lakes, color codes, fish sizes; <41 (etc.): walleye length less than 41 cm; RfD: reference dose for methyl mercury; other (yellow bars) include females beyond child-bearing age and males greater than age 14 years.

Methyl mercury exposure among individuals participating in the consumption survey is a function of fish size and lakes from which consumed walleye are harvested. When walleye are consumed without regard to the source lake, median and 95th percentile MeHg exposures (Figures 1(a) and 1(b)) are greatest for all groups when fish of 51 cm (20 inches) or larger are consumed; however, median exposure levels are less than the mercury reference dose (RfD) for all individuals and fish's sizes, while 95th percentile exposures are greater than the mercury RfD for children aged 1–5 consuming only fish larger than 41 cm (16 inches, Figures 1(a) and 1(b)). Median and 95th percentile MeHg exposure levels are greatest in young children aged 1–5 and 6–14 years (Figures 1(a) and 1(b)), and lower in women of child-bearing age and “other” individuals (men beyond aged 15 and women beyond child-bearing age, Figures 1(a) and 1(b)). 

**Figure 2:**
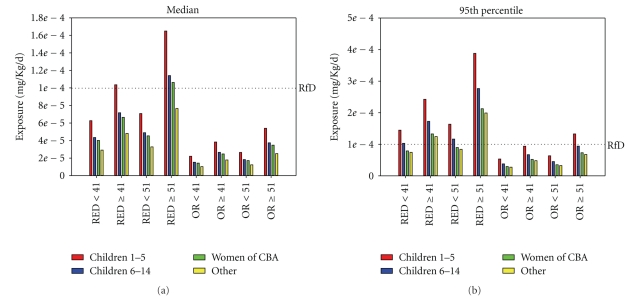
Median (a) and 95th percentile (b) exposure for consumption of fish harvested from color-coded red lakes (RED) and lakes color-coded other than red (OR), and for walleye less or greater than 41 and 51 cm. Other (yellow bars) include females beyond child-bearing age and males greater than age 14 years.

Median and 95th percentile MeHg exposures are highest when larger walleye (>51 cm) harvested from lakes that are color-coded red are consumed, and lower when walleye under 41 cm from nonred lakes are consumed (Figures 2(a) and 2(b)). Consumption of walleye at rates observed in the survey would result in exposure levels that are highest for children and lower for women of child bearing age and other adults; however, median exposure levels (Figure 2(a)) would only exceed the mercury reference for children aged 1–5 years who consume walleye greater than 41 cm harvested from color-coded red lakes (lakes containing walleye with the greatest MeHg concentrations), and for children aged 6–14 years and women of child-bearing age who consume fish larger than 51 cm from lakes that are color-coded red. While MeHg exposure patterns at the 95th percentile are similar to patterns at median exposure levels, 95th percentile exposures exceed the reference dose for MeHg when children and adults consume fish greater than 41 cm from color-coded red lakes, when children aged 1–5 and 6–14 years consume any fish from red lakes, and when children aged 1–5 consume fish greater than 51 cm (20 inches) from any lake (Figure 2(b)). 

The probabilistic analysis provides important information about the influence of lake source and walleye size on potential MeHg exposure among subgroups of tribal members and reinforces GLIFWC's approach to developing and disseminating walleye consumption advice. For example, the potential for median MeHg exposure to exceed the mercury reference dose among children and women of child-bearing age who consume only larger fish from red lakes (Figure 2(a)) is of concern. GLIFWC has developed and disseminated walleye consumption advice for these individuals: to not eat walleye larger than 51 cm (20 inches), to not eat walleye from lakes color-coded red, and to consume smaller walleye from lakes other than those color-coded red. The maps also encourage tribal members other than children and women of child-bearing age to eat fewer meals if they choose fish greater than 51 cm from any lake. Finally, the GLIFWC maps encourage tribal members to label and store walleye fillets so that consumption advice can be followed throughout the year by family members and others with whom walleye are shared. 

This analysis enhances GLIFWC's ability to focus and fine-tune its initiatives to protect the health of tribal members who harvest and consume walleye through its fish consumption advisory program. More importantly, it supports GLIFWC's age- and gender-based approach to exposure and risk reduction initiatives for tribal members while maintaining important tribal lifeways that include the harvest and consumption of walleye.

## Figures and Tables

**Table 1:** Percent of lakes with walleye meal frequency categories for the sensitive (women of child-bearing age and children under the age of 15 years) and general (all others) populations.

Advice category	Lake color code	Percent of lakes in each advice category	
		Sensitive population	General population
8 meals/month	Blue	0.3%	17.7%
4 meals/month	Green	2.7%	52.9%
2 meals/month	Yellow	24.6%	28.7%
1 meal/month	Orange	54.6%	0.7%
Do not eat	Red	17.8%	0.0%

**Table 2:** Methyl mercury concentrations in walleye (*n* = 221,960) from lakes for which GLIFWC issues consumption advice (see DeWeese et al. 2009 [9] for a description of lake codes and advice categories).

Lake Color	Walleye Size (cm)	Tissue Concenration (mg/kg)			
		Median	95th %	99th %	Max.
ALL	<41	0.11	0.40	0.51	0.91
ALL	≥41	0.22	0.62	0.95	3.10
ALL	<51	0.15	0.43	0.62	1.27
ALL	≥51	0.28	0.88	1.35	3.10
ALL	ALL	0.17	0.47	0.73	3.10
RED	<41	0.40	0.68	0.75	0.91
RED	≥41	0.61	1.09	1.87	3.10
RED	<51	0.43	0.74	0.89	1.27
RED	≥51	0.94	2.08	2.73	3.10
RED	ALL	0.44	0.80	1.11	3.10
OR	<41	0.11	0.31	0.45	0.71
OR	≥41	0.21	0.55	0.84	3.10
OR	<51	0.14	0.36	0.51	0.81
OR	≥51	0.28	0.80	1.20	3.10
OR	ALL	0.16	0.41	0.60	3.10

All: walleye harvested from all lakes for which GLIFWC issues consumption advice.

Red: walleye harvested from lakes color-coded red (those for which GLIFWC issues do-not-eat advice)

OR: walleye harvested from lakes color-coded other than red (lakes with consumption advice less restrictive than do-not-eat).

**Table 3:** Walleye Harvest Data, 2005–2007.

Total Walleye Harvested	221,960
Walleye with Length Information	120,836
Min Length (cm)	16.0
Max Length (cm)	86.1
Mean Length (cm)	41.1
Median Length (cm)	40.1
95th% Length (cm)	56.1
